# Ratios of involved nodes in early breast cancer

**DOI:** 10.1186/bcr934

**Published:** 2004-10-06

**Authors:** Vincent Vinh-Hung, Claire Verschraegen, Donald I Promish, Gábor Cserni, Jan Van de Steene, Patricia Tai, Georges Vlastos, Mia Voordeckers, Guy Storme, Melanie Royce

**Affiliations:** 1 Oncology Center, Academic Hospital, AZ-VUB, Vrije Universiteit Brussel, Jette, Belgium; 2 Student Management Healthcare Data, BISI-VUB, Computer Science and Medical Informatics, Vrije Universiteit Brussel, Jette, Belgium; 3 The University of New Mexico, Cancer Research and Treatment Center, Albuquerque, New Mexico, USA; 4 Decision Analyst, Burlington, Vermont, USA; 5 Surgical Pathology, Bács-Kiskun County Teaching Hospital, Kecskemét, Hungary; 6 Radiation Oncology, Saskatchewan Cancer Agency, Regina, Canada; 7 Gynecologic Oncology and Senology, Geneva University Hospitals, Geneva, Switzerland

**Keywords:** axillary lymph node ratio, breast neoplasm, functional form, loco-regional, log odds, nodal ratio, Nottingham Prognostic Index, predictive utility, prognostic factors, proportional hazards, proportion based, ratio-based prognostic index, SEER program, staging, survival

## Abstract

**Introduction:**

The number of lymph nodes found to be involved in an axillary dissection is among the most powerful prognostic factors in breast cancer, but it is confounded by the number of lymph nodes that have been examined. We investigate an idea that has surfaced recently in the literature (since 1999), namely that the proportion of node-positive lymph nodes (or a function thereof) is a much better predictor of survival than the number of excised and node-positive lymph nodes, alone or together.

**Methods:**

The data were abstracted from 83,686 cases registered in the Surveillance, Epidemiology, and End Results (SEER) program of women diagnosed with nonmetastatic T1–T2 primary breast carcinoma between 1988 and 1997, in whom axillary node dissection was performed. The end-point was death from breast cancer. Cox models based on different expressions of nodal involvement were compared using the Nagelkerke R^2 ^index (R^2^_N_). Ratios were modeled as percentage and as log odds of involved nodes. Log odds were estimated in a way that avoids singularities (zero values) by using the empirical logistic transform.

**Results:**

In node-negative cases both the number of nodes excised and the log odds were significant, with hazard ratios of 0.991 (95% confidence interval 0.986–0.997) and 1.150 (1.058–1.249), respectively, but without improving R^2^_N_. In node-positive cases the hazard ratios were 1.003–1.088 for the number of involved nodes, 0.966–1.005 for the number of excised nodes, 1.015–1.017 for the percentage, and 1.344–1.381 for the log odds. R^2^_N _improved from 0.067 (no nodal covariate) to 0.102 (models based on counts only) and to 0.108 (models based on ratios).

**Discussion:**

Ratios are simple optimal predictors, in that they provide at least the same prognostic value as the more traditional staging based on counting of involved nodes, without replacing them with a needlessly complicated alternative. They can be viewed as a per patient standardization in which the number of involved nodes is standardized to the number of nodes excised. In an extension to the study, ratios were validated in a comparison with categorized staging measures using blinded data from the San Jose–Monterey cancer registry. A ratio based prognostic index was also derived. It improved the Nottingham Prognostic Index without compromising on simplicity.

## Introduction

Breast cancer is the most common neoplasm in women. Nodal status as determined by pathologic examination of lymph nodes has repeatedly been shown to be the single most important predictor of survival in breast cancer [1]. The absolute number of pathologically involved nodes has also been shown to be an important prognostic factor in breast cancer survival [2-6]. The extent of lymph node involvement is incorporated into prognostic indices such as the Nottingham Prognostic Index [7-9] (see Additional files 1,2,3,4,5,6). Old lymph node stage measures categorized cases according to whether they had none, one to three, or four or more involved nodes, and recently according to more detailed subdivisions [10,11] (see Additional files 2,3,4,5 and 7).

However, several authors have noted the inherent confounding by the number of excised nodes [12,13]. To address the variability of nodal examination, an intuitive approach is to use the proportion or the percentage of involved nodes, as was suggested by Rostgaard and coworkers [14]. The proportion can immediately be derived from pathology reports that clearly state the total number of lymph nodes examined and the total number of involved nodes [1,15]. The proportion has received increasing attention in the literature, providing a reference base on which its clinical relevance may be discussed [13,14,16-27].

**Figure 1:**
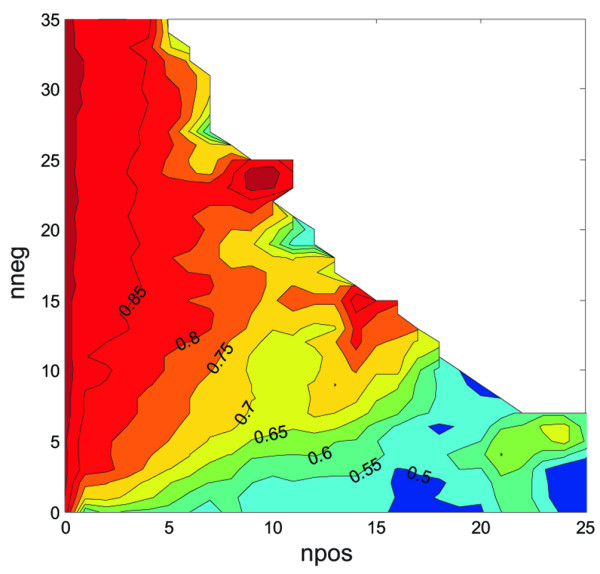
Joint effect of the numbers of involved nodes (npos) and uninvolved nodes (nneg) on survival in T1–T2 breast cancer. Part of the contour plot was partially filled at the corners by padding. The pattern of isoprobability contours radiating from the origin suggests that similar ratios of involved/uninvolved nodes were associated with similar Kaplan–Meier survival estimates (for example. 8 npos/10 nneg has approximately the same 75% [contour line 0.75] 5-year survival chance as 4 npos/5 nneg). Reproduced with permission from Vin-Hung and coworkers [28]. Colors were omitted in the original publication.

In this report the modeling utility of the proportion of involved nodes is compared with the absolute numbers of involved nodes and of examined nodes. There is a one-to-one correspondence between proportion and ratio between involved and uninvolved nodes. A previous study hinted at an apparent linear relationship with survival between involved and uninvolved nodes (Fig. 1) [28], and therefore this report also examines the utility of expressing ratios as odds instead of proportions.

The absolute numbers considered in the study were the number of nodes examined (excised; nx), the number of involved nodes (np), and the number of uninvolved nodes (nn).

## Methods

The SEER (Surveillance, Epidemiology, and End Results) program of the USA [29] provides extensive cancer incidence data from 11 population-based registries. The data used in the present study were extracted from nine of those registries: San Francisco-Oakland, Connecticut, Metropolitan Detroit, Hawaii, Iowa, New Mexico, Seattle (Puget Sound), Utah, and Metropolitan Atlanta.

Selected patients were women without a previous history of cancer who presented with a noninflammatory invasive breast carcinoma, which was histologically confirmed and diagnosed between 1988 and 1997, with specified tumor size no larger than 50 mm (T1 and T2), strictly confined to breasts without distant metastasis, and in which curative surgery and axillary lymph node dissection were performed with removal of at least one node. Cases with involvement of skin, hypodermis or pectoral muscles, or with deep fixation were excluded. Patients who had undergone subcutaneous mastectomy, radical mastectomy, or preoperative or intraoperative radiotherapy were excluded. Data on systemic treatment were not available and therefore could not be taken into account. Certain records were rejected because of data quality concerns: uncertain sequence of treatment, nonhospital based data records, month of diagnosis unknown, or race unknown. Examination of outliers (scarce and extreme values) resulted in further exclusion of cases with more than 50 nodes examined, '0 months' of follow up, and age at diagnosis under 25 years or older than 95 years.

The follow-up cut-off date was 31 December 1999. The survival end event was defined as death from breast cancer.

The proportions of involved nodes were expressed as percentages ([np/nx] × 100%). The log odds of nodal involvement were computed using the empirical logistic transform: L = Log_e_([np + 0.5]/ [nn + 0.5]) [30]. The transform, also called the sample logit, avoids singularities caused by null observations, and is the least biased estimator of the true log odds [31]. (Note that, with hindsight, Fig. 1 shows a logarithmic relationship.) Unadjusted mortality (the number of patients who died divided by the number of patients at risk) as a function of the ratios was used for descriptive purposes.

The utilities of the percentage and log odds were evaluated in different multivariate Cox proportional hazards models [32]. The numbers np and nx, the percentage (np/nx) × 100%, and the L transform were entered as quantitative continuous variables in different combinations. The corresponding hazard ratios were each time computed within a Cox model that included tumor size, age at diagnosis, and year of diagnosis modeled as quantitative continuous variables; and the registry area, race, marital status, tumor topography, histologic type and grade, estrogen and progesterone receptor status, type of primary surgery, and administration of postoperative radiotherapy modeled as qualitative variables. The qualitative variables were converted or expanded as needed into dummy variables to allow binary coding ('married' versus 'not married', 'high grade' versus 'not high grade', for example, and so on). A first order interaction between type of surgery and postoperative radiotherapy was included for consistency with a previous analysis [33]. The models were computed in all cases irrespective of nodal status, and then as a function of positive or negative nodal status. The functional forms were assessed using the generalized additive model procedure [34].

The Nagelkerke R^2 ^index (R^2^_N_) was used to score the different Cox models [35]. R^2 ^represents the proportion of variation explained by covariates in regression models [35-37]. R^2^_N _divides R^2 ^by its maximum attainable value to scale it to within the range 0–1. R^2^_N _is close to 1 for a perfectly predictive model, and close to 0 for a model that does not discriminate between short and long survival times.

Statistical analyses were performed using Splus (Insightful Corporation, Seattle, WA, USA) statistical software.

## Results

In the 2002 SEER release [29], 188,410 women were diagnosed with breast tumors from 1988 to 1997, of whom 132,457 had a hospital based histopathologic diagnosis of unilateral invasive carcinoma. A total of 83,686 cases matched the selection criteria; 58,070 were node-negative and 25,616 node-positive. The median follow-up time was 73 months (range 1–143 months) for patients still alive at the follow-up cut-off date (31 December 1999). Characteristics of the patients were presented elsewhere [33]. Except for some additional cases due to updated registration minus the exclusion of outliers resulting in 90 fewer cases, there were no noticeable differences in the distribution of the characteristics.

The median number of nodes examined was 15 (range 1–50, mean ± standard deviation 15.4 ± 6.5). Among the node-positive patients, the median number of involved nodes was 2 (1–46, 4.1 ± 4.8).

**Figure 2:**
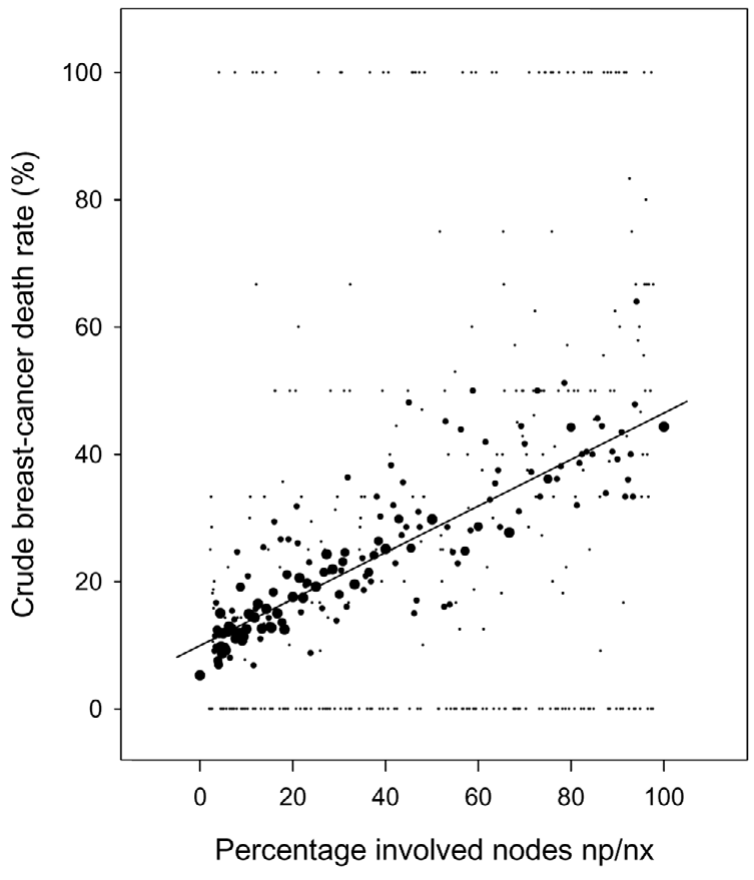
Unadjusted breast-cancer mortality as a function of the percentage of involved nodes in T1–T2 breast cancer based on the SEER (Surveillance, Epidemiology, and End Results) program data. Dot size computed as a step function of the number of patients at risk: smallest dots 1–20 patients and the largest dots >200 patients. The straight line highlights the trend but should not be interpreted as the basis for extrapolation.

Table 1 shows the distribution of the percentages of involved nodes. Figure 2 is a plot of the corresponding breast cancer mortality, which appears to increase linearly with the np/nx percentage.

**Figure 3:**
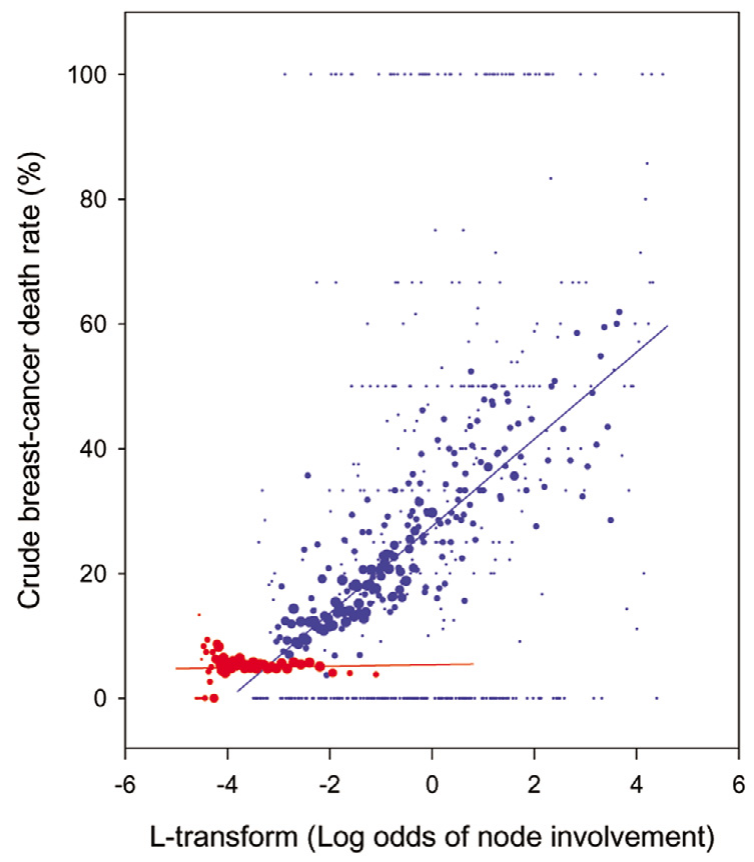
Unadjusted breast cancer mortality as a function of the estimated log odds of nodal involvement in T1–T2 breast cancer. Red dots are node-negative patients, and blue are node-positive patients. The smallest dots represent 1–20 patients and the largest dots represent >200 patients. The straight lines highlight the different slopes but should not be interpreted as the basis for extrapolation (they would extrapolate to <0% or >100% mortalities).

Table 2 shows the distribution of the log odds for nodal involvement. Figure 3 plots the corresponding breast cancer mortality. There is an initial, almost flat segment for values of L ≤ -3, which is followed by a steeply sloping upward segment. The initial flat segment corresponds mostly to node-negative cases. The sloping upward segment corresponds to node-positive cases, with more positive L values indicating more involved nodes and/or fewer uninvolved nodes. There is an overlap between node-negative and node-positive cases for L values between -3.5 and -1.

**Figure 4:**
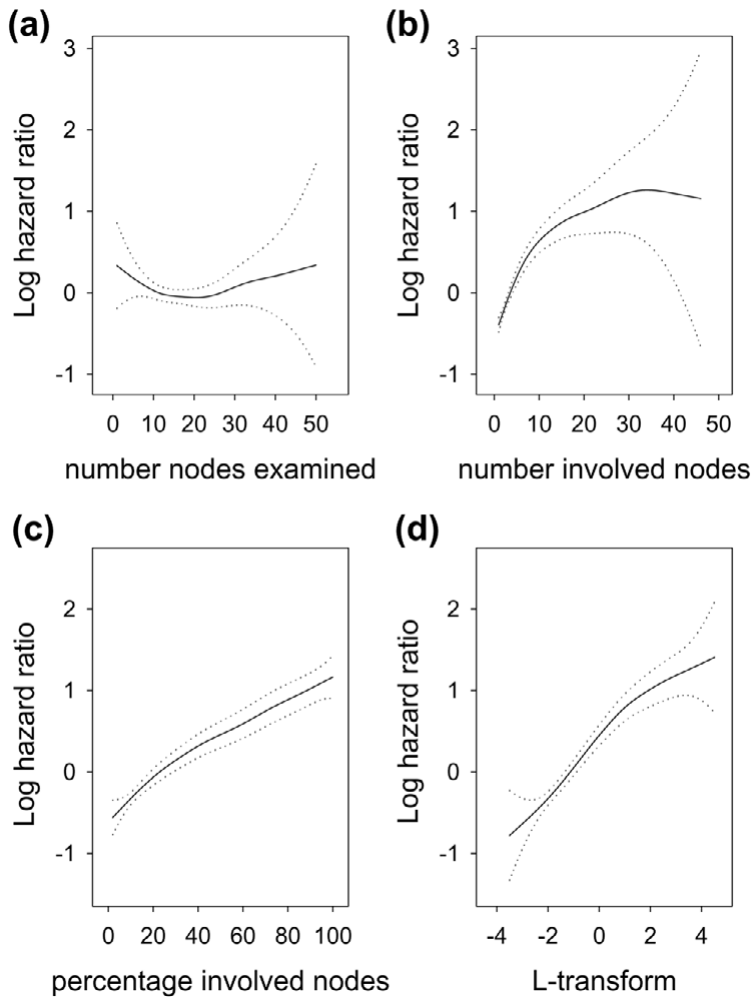
Adjusted breast cancer mortality in T1–T2 node-positive breast cancer as a function of **(a) **number of nodes examined, **(b) **number of involved nodes, **(c) **percentage of involved nodes, and **(d) **log odds of involved nodes. Dotted lines indicate the 95% confidence interval. Plots are based on multivariate models that included all non-nodal covariates listed in the Methods section and a single nodal covariate. Analysis of cases pooled irrespective of nodal status revealed a more marked nonlinearity in plot c between 0% and 20% involved nodes. Otherwise, different combinations of nodal covariates gave similar plots.

In multivariate analyses, np and nx exhibited marked nonlinearity and widely diverging confidence intervals (Fig. 4a,4b). The linearity improved for the percentage (np/nx) × 100% and the L transform, which also showed more homogeneously distributed confidence intervals (Fig. 4c,4d).

The upper section of Table 3 shows a comparison between proportional hazards models that included different combinations of np, nx, np/nx, and L for all patients, irrespective of nodal status. Based on R^2^_N_, the best predictive covariate was L (model 6), with a small improvement contributed by nx (model 10). In the model with nx alone (model 3), nx was statistically significant but its contribution to global model fit appeared negligible because the R^2^_N _did not change from the baseline 0.069 (model 1). The contribution of np alone was substantial, with a change of R^2^_N _from baseline 0.069 to 0.093 (model 2). However, adding np and/or nx onto L or onto np/nx provided no improvement, except in the already mentioned model 10.

The middle section of Table 3 shows multivariate analysis performed for node-positive cases only. Models based on separately expressed numbers provided the lowest R^2^_N _(models 2–4). The largest R^2^_N _values were all observed in models incorporating L or np/nx (models 5–11). The simplest model appeared to be based on np/nx alone (model 5; R^2^_N _= 0.108). A small improvement was contributed by np (model 7; R^2^_N _= 0.109).

The lower section of Table 3 shows the analysis performed for node-negative cases only. Because np, by definition, equals 0, there are only four models. They show that nx (model 3) and L (model 6) are statistically significant, but these variables either alone or in combination did not improve the index R^2^_N_.

The multivariate computations were also performed by considering death from any cause as the end-point. There were no notable discrepancies.

## Discussion

Although many data were evaluated in the present study, there are weaknesses. The data are heterogeneous. Histopathologic characteristics such as grade could not be verified. Neoadjuvant systemic treatment might have modified the yield of nodes [38]. Important information such as how patients were selected for any particular treatment is missing. Undocumented comorbidity might have affected the extent of nodal dissection. An imbalance in the delivery of chemotherapy or hormone therapy could have affected the distribution of deaths. For all of these reasons, the present results should be considered explorative and must be validated independently.

Since about 1999 a growing number of studies have investigated nodal ratios. In the studies that compared the numbers of involved nodes with ratios in multivariate models, the majority found that ratios were better than numbers as prognostic indicators [13,16,18,19,24,26]. Ratios (expressed as percentages or log odds) have a better prognostic impact than do isolated numbers and, unlike numbers, they are not associated with inconsistent findings. Part of the explanation might be that a ratio can be interpreted as a form of standardization in which the number of involved nodes found in a patient is standardized to the number of nodes examined in that same patient [20]. It is noteworthy that the hazard ratios for np/nx were almost unaffected by the model (column np/nx [%] in Table 3), whereas the hazard ratios for np and/or nx exhibited more variability (columns np and nx in Table 3).

As a prognostic factor, np/nx appears the most convenient. Figure 2 shows that, for node-positive cases presenting with 0–10% involved nodes, the crude breast cancer mortality risk for an average follow up of 6 years is about 5%, and with 90–100% involved nodes the mortality is about 45%. For any intermediate value for the percentage of node involvement, the mortality risk is easily interpolated.

The estimated log odds L provided results very similar to those with np/nx. Overall, L improves on np/nx when all cases are considered together (column L in Table 3). The log odds appears useful for integrating node-negative and node-positive cases while avoiding more complex modeling, which we performed previously [39]. However, there is a range of L values in which node-negative and node-positive cases overlap (Table 2, Figure 3). In an analysis of all cases pooled, the overlap might blur the prognostic difference between node-negative status based on a very small number of excised nodes, and node-positive status based on a large number of excised nodes but with few involved nodes. The literature on the log odds of node involvement is scarce, and the utility of the L transform needs independent confirmation.

The present findings indicate that the favorable survival attributed to higher numbers of nodes removed, as suggested by Krag and Single [40], might be due to different model specifications. The number of patients is huge and statistical significance can easily be demonstrated but without necessarily implying any major clinical impact. Undoubtedly, the uncertainty about node negativity increases when nx (the number of excised nodes) is small. However, the predictive utility attributable to nx is exceedingly small (Table 3, lower section). This dissociation between statistical significance and predictive utility appears counterintuitive. Nevertheless, it is in keeping with findings from Fisher and coworkers [41], who noted that prognosis was unaffected by the number of excised nodes when nodal status was reported to be negative. This is also supported by a recent report based on 3800 patients [42] in which the number of excised nodes was predictive of the risk for recurrence in node-positive but not in node-negative patients.

Sentinel node biopsy has gained wide acceptance since 1997 and it is used to determine the need for axillary dissection [43]. Because our selection of patients was from 1988 until 1997, it is unlikely that sentinel nodes could have represented any substantial part of the present study. The prognostic impact of one involved node in patients who had one node removed in this study cannot be extrapolated to the patient found with one involved node in a sentinel node procedure. However, in the prediction of nonsentinel node involvement when one or more sentinel nodes are found to be involved, Cserni and coworkers [44] reported that the number of sentinel nodes and the percentage of positive sentinel nodes were jointly significant predictors. A closely related finding that also highlights the predictive role of ratios was reported in a recent Australian study [45], in which the prediction model was determined by patient age, by the number of sentinel nodes, and by the proportion of involved sentinel nodes.

## Conclusion

We found the percentage of involved nodes to be the most directly useful indicator of nodal involvement, but this is limited to node-positive cases. The log odds of nodal involvement performed equally well in node-positive and node-negative patients. The log odds might provide a unified approach to the modeling of nodal involvement. The present results and the growing literature argue that ratios should be considered in the staging of axillary dissection.

## Competing interests

The author(s) declare that they have no completing interests.

## Abbreviations

L = empirical logistic transform (estimated log odds); nn = number of axillary lymph nodes free from tumor involvement; np = number of pathologically involved axillary lymph nodes; nx = number of axillary lymph nodes examined (excised); R^2^_N _= Nagelkerke R^2 ^index.

## Supplementary Material

Additional File 1 Text on deriving a ratio based prognostic index. Click here for file

Additional File 2 Table evaluating nodal staging measures using breast cancer data from the San Jose–Monterey registry: all cases irrespective of nodal status. Click here for file

Additional File 3 Table evaluating nodal staging measures using breast cancer data from the San Jose–Monterey registry: node-positive patients. Click here for file

Additional File 4 Table providing a simulation of small datasets of 300 breast cancer patients, irrespective of nodal status. Click here for file

Additional File 5 Table providing a simulation of small datasets of 300 node-positive breast cancer patients Click here for file

Additional File 6 Figure showing Kaplan–Meier survival estimates for T1–T2 breast cancer abstracted from the San Jose–Monterey registry. Click here for file

Additional File 7 Text on ratios and the TNM categorization. Click here for file

## Figures and Tables

**Table 1:** Distribution of the percentages of involved nodes and corresponding unadjusted mortality

Percentage of involved nodes (np/nx [%])	Number of patients at risk	All-cause mortality (%)	Breast cancer mortality (%)	Non-breast-cancer mortality (%)
0	58070	15.9	5.2	10.7
1–10	8695	21.6	11.7	9.9
11–20	6350	26.3	15.7	10.6
21–30	3003	30.8	19.9	10.9
32–40	2055	33.1	22.7	10.4
41–50	1487	41.0	29.6	11.4
51–60	889	37.8	28.2	9.6
61–70	719	47.0	33.7	13.4
71–80	717	50.3	40.3	10.0
81–90	658	52.6	40.4	12.2
91–100	1043	58.6	45.2	13.4

np, number of pathologically involved axillary lymph nodes; nx, number of axillary lymph nodes examined (excised).

**Table 2:** Distribution of the estimated log odds of nodal involvement, and corresponding unadjusted mortality

L	All patients			Node-negative patients			Node-positive patients		
	
	Number of patients at risk	Breast cancer mortality	Non-breast-cancer mortality	Number of patients at risk	Breast cancer mortality	Non-breast-cancer mortality	Number of patients at risk	Breast cancer mortality	Non-breast-cancer mortality
L ≤ -4	3053	5.4	9.2	3053	5.4	9.2	0		
-4 < L ≤ -3	44695	5.3	10.2	44503	5.2	10.2	192	13.5	7.8
-3 < L ≤ -2	17619	8.0	11.5	10187	5.4	12.8	7432	11.7	9.7
-2 < L ≤ -1	9173	15.4	10.9	327	4.0	19.3	8846	15.8	10.6
-1 < L ≤ 0	5120	24.0	11.0	0			5120	24.0	11.0
0 < L ≤ 1	1943	31.9	11.3	0			1943	31.9	11.3
1 < L ≤ 2	1113	40.3	11.1	0			1113	40.3	11.1
2 < L ≤ 3	536	44.4	13.8	0			536	44.4	13.8
3 < L ≤ 4	376	47.6	13.0	0			376	47.6	13.0
4 < L	58	56.9	10.3	0			58	56.9	10.3

L = empirical logistic transform (estimated log odds).

**Table 3:** Comparison of models

Patients studied	R^2^_N_	Hazard ratios (95% confidence interval)			
			
		np	nx	np/nx (%)	L
All cases					
1: no nodal variables	0.069				
2: np	0.093	1.093 (1.090–1.097)			
3: nx	0.069		0.998 (0.995–1.001)		
4: np, nx	0.096	1.112 (1.108–1.116)	0.970 (0.966–0.974)		
5: np/nx (%)	0.104			1.023 (1.022–1.024)	
6: L	0.108				1.459 (1.442–1.477)
7: np, np/nx (%)	0.104	1.010 (1.004–1.017)		1.021 (1.020–1.023)	
8: nx, np/nx (%)	0.104		1.002 (0.999–1.005)	1.023 (1.022–1.024)	
9: np, L	0.108	0.998 (0.992–1.005)			1.466 (1.435–1.497)
10: nx, L	0.109		1.009 (1.006–1.013)		1.462 (1.445–1.479)
11: np, nx, np/nx (%)	0.104	1.013 (1.004–1.022)	0.998 (0.994–1.002)	1.021 (1.019–1.022)	
12: np, nx, L	0.109	0.967 (0.957–0.976)	1.021 (1.017–1.026)	.	1.599 (1.554–1.646)
Node-positive patients					
1: no nodal variables	0.067				
2: np	0.095	1.066 (1.062–1.071)			
3: nx	0.067		0.996 (0.992–1.000)		
4: np, nx	0.102	1.088 (1.083–1.093)	0.966 (0.961–0.971)		
5: np/nx (%)	0.108			1.017 (1.016–1.018)	
6: L	0.108				1.379 (1.355–1.403)
7: np, np/nx (%)	0.109	1.013 (1.006–1.020)		1.015 (1.014–1.017)	
8: nx, np/nx (%)	0.108		1.005 (1.001–1.009)	1.017 (1.016–1.018)	
9: np, L	0.108	1.008 (1.001–1.016)			1.344 (1.306–1.384)
10: nx, L	0.108		1.005 (1.001–1.009)		1.381 (1.357–1.405)
11: np, nx, np/nx (%)	0.109	1.016 (1.005–1.028)	0.998 (0.991–1.004)	1.015 (1.013–1.017)	
12: np, nx, L	0.108	1.003 (0.990–1.017)	1.003 (0.996–1.010)		1.366 (1.304–1.431)
Node-negative patients					
1: no nodal variables	0.045	NA			
3: nx	0.045	NA	0.991 (0.986–0.997)		
6: L	0.045	NA			1.150 (1.058–1.249)
10: nx, L	0.045	NA	1.001 (0.984–1.019)		1.169 (0.902–1.514)

Shown are all nodal status combined, and node-positive patients and node-negative patients separately. A hazard ratio >1 indicates increased risk for death from breast cancer. All models are multivariate, adjusting for the effect of covariates listed in the Methods section: tumor size, age at diagnosis, year of diagnosis, registry area, race, marital status, tumor topography, histologic type and grade, estrogen and progesterone receptor status, type of primary surgery, and administration of postoperative radiotherapy. NA, not applicable; np, number of involved nodes; nx, number of nodes examined; np/nx (%), percentage of nodes involved; L, log-odds of node involvement; R^2^_N_, Nagelkerke R^2 ^index of global model fit (0 = lack of fit, 1 = perfect fit).
