# Supplementary material for: Ratios of involved nodes in early breast cancer
Source: Breast Cancer Res. 2004 Oct 6;6(6):R680–8. doi: 10.1186/bcr934 (PMC1064081; doi:10.1186/bcr934)
Supplement: Additional File 1 — Text on deriving a ratio based prognostic index. [file bcr934-S1.doc]

# Additional file 1: Deriving a ratio-based prognostic index

The Nottingham Prognostic Index (NPI) combines tumour size, histological grade and extent of lymph node involvement in a single index [1]. The NPI is calculated using the following equation:

NPI = 0.2 x tumour size_(cm)_ + grade_(1−3)_ + nodal score_(1 if_ **_np_**_=0, 2 if_ **_np_**_≤3, 3 if_ **_np_**_>3)_

The NPI is usually categorized into 3 levels, low risk (NPI<3.4), moderate risk (3.4≤NPI<5.4), and high risk (NPI≥5.4) [2].

This Additional file addresses whether or not ratios could improve the NPI.

The search for a ratio-based index was constrained by the following conditions: same components (tumour size, grade, nodal score); reasonable partition of the data irrespective of nodal status; simplicity.

A tentative solution derived from the main manuscript's data was:

*Lpi* = 0.2 x tumour size_(cm)_ + 0.5 x high-grade_(1 if high grade, 0 otherwise)_ + 0.25 x ***L*** _(as-is)_

recalling that ***L*** is the log-odds of node involvement, computed as

***L*** = Log_e_ [(number of involved nodes + 0.5)/(number of examined nodes + 0.5)]

where Log_e_ is the natural logarithm.

Three categorized levels of the *Lpi* (log-odds prognostic index) were defined using 0 and 1 cutoffs: *Lpi* ≤ 0, 0< *Lpi* ≤ 1, and *Lpi* > 1.

The verification used the San Jose-Monterey data and sampling methods identical to Additional file 1, except that the baseline models did not include tumor size and grade which were already incorporated in the indexes.

Results are shown in Additional files 3-6, models B. The categorized NPI showed in all four groups of data a better model fit than the categorized *Lpi*. However, the separate NPI levels displayed wide confidence intervals (Additional files 3 and 4). In the node-positive subsamples, the moderate and high risk NPI levels (relatively to the low-risk NPI level) rarely reached statistical significance (Additional files 5 and 6). By contrast, the categorized *Lpi* displayed a good stability. Its separate categorization could also cause spurious non-significance but less than other staging measures.

Incorporating the indexes into multivariate models are useful to verify their properties, but defeat their purpose to simplify the modeling. The indexes were therefore examined by Kaplan-Meier survival curves without adjustment. Additional file 7 shows the plots according to index. Both the NPI and the *Lpi* tested significant in all graphs. However, the separation between the different prognostic levels was clearer with the *Lpi* than with the NPI.

The development of the NPI was originally based on a multivariate survival analysis. It was a stroke of genius when Haybittle et al went beyond the crude results of the analysis and used simplified coefficients [3]. The present manuscript attempted to emulate their approach. The results suggest that a ratio-based index might improve the NPI without compromising on its original simplicity.

### References

1. Kollias J, Vernon-Roberts E, Blamey RW, Elston CW: **A simple index to predict prognosis independent of axillary node information in breast cancer: comment.** *Aust N Z J Surg* 1998, **68:**865-866.

2. Rostgaard K, Mouridsen HT, Vaeth M, Holst H, Olesen KP, Lynge E: **A modified Nottingham prognostic index for breast cancer patients diagnosed in Denmark 1978–1994.** *Acta Oncol* 2001, **40:**838-843.

3. Haybittle JL, Blamey RW, Elston CW, Johnson J, Doyle PJ, Campbell FC, Nicholson RI, Griffiths K: **A prognostic index in primary breast cancer.** *Br J Cancer* 1982, **45:**361-366.
