# Supplementary material for: Ratios of involved nodes in early breast cancer
Source: Breast Cancer Res. 2004 Oct 6;6(6):R680–8. doi: 10.1186/bcr934 (PMC1064081; doi:10.1186/bcr934)
Supplement: Additional File 2 — Table evaluating nodal staging measures using breast cancer data from the San Jose–Monterey registry: all cases irrespective of nodal status. [file bcr934-S2.doc]

**Additional file 2**

Evaluation of nodal staging measures using breast-cancer data from the San Jose-Monterey registry. All cases irrespective of nodal status, 4204 patients. A: ratio-based models compared with TNM. B: Nottingham Prognostic Index (NPI)-based and log-odds prognostic index *(Lpi)*-based models.

| ***(All cases)*** | **R^2^_N_** | **Hazard ratio** | **95% confidence interval** |
| --- | --- | --- | --- |
| **A.** |  |  |  |
| Baseline model (no nodal variable) | 0.093 |  |  |
| Model with TNM nodal staging | 0.116 |  |  |
| N0 (np=0) |  | 1 |  |
| N1 (0<np ≤3) |  | 1.541 | (1.274-1.863) |
| N2 (3<np ≤9) |  | 2.524 | (1.984-3.210) |
| N3 (np>9) |  | 4.206 | (3.117-5.675) |
| Model with categorized proportion of involved nodes | 0.116 |  |  |
| p0 (0%) |  | 1 |  |
| p1 (1-20%) |  | 1.501 | (1.234-1.824) |
| p2 (21-50%) |  | 2.393 | (1.868-3.064) |
| p3 (51-100%) |  | 3.703 | (2.844-4.824) |
| Model with categorized log-odds of involved nodes | 0.115 |  |  |
| Ln0 (L ≤−3.5) |  | 1 |  |
| Ln1 (−3.5<L ≤−1) |  | 1.493 | (1.225-1.818) |
| Ln2 (−1< L ≤0) |  | 2.758 | (2.040-3.730) |
| Ln3 (L>0) |  | 4.257 | (3.158-5.739) |
| Model with simple proportion of involved nodes | 0.114 | 1.017 | (1.014-1.020) |
| Model with estimated log-odds of involved nodes | 0.116 | 1.315 | (1.251-1.381) |
| **B.** |  |  |  |
| Baseline reduced model (no tumour size, no grade, no nodal variable) | 0.068 |  |  |
| Model with categorized Nottingham prognostic index NPI | 0.107 |  |  |
| low (NPI<3.4) |  | 1 |  |
| moderate (3.4≤NPI<5.4) |  | 1.651 | (1.372-1.988) |
| high (NPI≥5.4) |  | 4.407 | (3.537-5.492) |
| Model with categorized ratio-based index Lpi | 0.102 |  |  |
| L0 (Lpi ≤0) |  | 1 |  |
| L1 (0 <Lpi ≤1) |  | 2.190 | (1.858-2.581) |
| L2 (Lpi >1) |  | 4.826 | (3.656-6.370) |
