# Supplementary material for: Ratios of involved nodes in early breast cancer
Source: Breast Cancer Res. 2004 Oct 6;6(6):R680–8. doi: 10.1186/bcr934 (PMC1064081; doi:10.1186/bcr934)
Supplement: Additional File 3 — Table evaluating nodal staging measures using breast cancer data from the San Jose–Monterey registry: node-positive patients. [file bcr934-S3.doc]

## Additional file 3

Evaluation of nodal staging measures using breast-cancer data from the San Jose-Monterey registry. Node-positive status, 1243 patients. A: ratio-based models compared with TNM. B: Nottingham Prognostic Index (NPI)-based and log-odds prognostic index *(Lpi)*-based models.

| ***(Node-positive)*** | **R^2^_N_** | **Hazard ratio** | **95% confidence interval** |
| --- | --- | --- | --- |
| **A.** |  |  |  |
| Baseline model (no nodal variable) | 0.054 |  |  |
| Model with TNM nodal categories | 0.144 |  |  |
| N1 (0<np ≤3) |  | 1 |  |
| N2 (3<np ≤9) |  | 1.645 | (1.265-2.140) |
| N3 (np>9) |  | 2.743 | (1.991-3.779) |
| Model with categorized proportion of involved nodes | 0.145 |  |  |
| p1 (1-20%) |  | 1 |  |
| p2 (21-50%) |  | 1.598 | (1.218-2.097) |
| p3 (51-100%) |  | 2.572 | (1.922-3.443) |
| Model with categorized log-odds of involved nodes | 0.143 |  |  |
| Ln1 (−3.5<L ≤−1) |  | 1 |  |
| Ln2 (−1< L ≤0) |  | 1.530 | (1.149-2.037) |
| Ln3 (L>0) |  | 2.447 | (1.839-3.255) |
| Model with simple proportion of involved nodes | 0.145 | 1.014 | (1.010-1.018) |
| Model with estimated log-odds of involved nodes | 0.144 | 1.288 | (1.193-1.390) |
| **B.** |  |  |  |
| Baseline reduced model (no tumour size, no grade, no nodal variable) | 0.042 |  |  |
| Model with categorized Nottingham prognostic index NPI | 0.126 |  |  |
| low (NPI<3.4) |  | 1 |  |
| moderate (3.4≤NPI<5.4) |  | 1.509 | (0.615-3.702) |
| high (NPI≥5.4) |  | 3.326 | (1.358-8.145) |
| Model with categorized ratio-based index Lpi | 0.121 |  |  |
| L0 (Lpi ≤0) |  | 1 |  |
| L1 (0 <Lpi ≤1) |  | 1.780 | (1.319-2.402) |
| L2 (Lpi >1) |  | 3.330 | (2.304-4.812) |
