# Supplementary material for: Ratios of involved nodes in early breast cancer
Source: Breast Cancer Res. 2004 Oct 6;6(6):R680–8. doi: 10.1186/bcr934 (PMC1064081; doi:10.1186/bcr934)
Supplement: Additional File 4 — Table providing a simulation of small datasets of 300 breast cancer patients, irrespective of nodal status. [file bcr934-S4.doc]

## Additional file 4

Simulation of small datasets of 300 breast cancer patients, irrespective of nodal status. Summary of modeling performed on 1000 random subsamples ("runs") extracted from the San Jose-Monterey registry. Significance level *P*-value 0.05. A: ratio-based models compared with TNM. B: Nottingham Prognostic Index (NPI)-based and log-odds prognostic index *(Lpi)*-based models.

| **Model**  ***(All cases)*** | **mean R^2^_N_** | **Number of runs in which the nodal variable contributed significantly to global model**  **(Likelihood ratio test)** | **Number of runs in which the "N1" vs. "N0" category, or the continuous variable, was found significant**  **(Wald test)** | **Number of runs in which the "N2" vs. "N0" category was found significant**  **(Wald test)** | **Number of runs in which the "N3" vs. "N0" category was found significant**  **(Wald test)** |
| --- | --- | --- | --- | --- | --- |
| **A.** |  |  |  |  |  |
| no nodal variable | 0.155 |  |  |  |  |
| TNM categories | 0.191 | 613 | 250 | 492 | 627 |
| Categorized proportion | 0.192 | 626 | 220 | 438 | 676 |
| Categorized log-odds | 0.190 | 599 | 157 | 415 | 673 |
| Proportion | 0.181 | 703 | 739 |  |  |
| Estimated log-odds | 0.183 | 741 | 764 |  |  |
| **B.** |  |  |  |  |  |
| no tumour size, no grade, no nodal variable | 0.119 |  |  |  |  |
| Categorized NPI | 0.169 | 867 | 282 | 918 |  |
| Categorized Lpi | 0.164 | 822 | 688 | 733 |  |
