# Supplementary material for: Ratios of involved nodes in early breast cancer
Source: Breast Cancer Res. 2004 Oct 6;6(6):R680–8. doi: 10.1186/bcr934 (PMC1064081; doi:10.1186/bcr934)
Supplement: Additional File 5 — Table providing a simulation of small datasets of 300 node-positive breast cancer patients [file bcr934-S5.doc]

## Additional file 5

Simulation of small datasets of 300 node-positive breast cancer patients. Summary of modeling performed on 1000 random subsamples ("runs") extracted from the San Jose-Monterey registry. Significance level *P*-value 0.05. A: ratio-based models compared with TNM. B: Nottingham Prognostic Index (NPI)-based and log-odds prognostic index *(Lpi)*-based models.

| **Model**  ***(Node-positive)*** | **mean R^2^_N_** | **Number of runs in which the nodal variable contributed significantly to global model**  **(Likelihood ratio test)** | **Number of runs in which the "N2" vs. "N1" category, or the continuous variable, was found significant**  **(Wald test)** | **Number of runs in which the "N3" vs. "N1" category was found significant**  **(Wald test)** |
| --- | --- | --- | --- | --- |
| **A.** |  |  |  |  |
| no nodal variable | 0.167 |  |  |  |
| TNM categories | 0.200 | 749 | 439 | 799 |
| Categorized proportion | 0.202 | 773 | 377 | 829 |
| Categorized log-odds | 0.200 | 744 | 317 | 815 |
| Proportion | 0.198 | 833 | 846 |  |
| Estimated log-odds | 0.197 | 816 | 831 |  |
| **B.** |  |  |  |  |
| no tumour size, no grade, no nodal variable | 0.135 |  |  |  |
| Categorized NPI | 0.177 | 858 | 22 | 30 |
| Categorized Lpi | 0.172 | 814 | 464 | 863 |
