# Supplementary material for: Ratios of involved nodes in early breast cancer
Source: Breast Cancer Res. 2004 Oct 6;6(6):R680–8. doi: 10.1186/bcr934 (PMC1064081; doi:10.1186/bcr934)
Supplement: Additional File 6 — Figure showing Kaplan–Meier survival estimates for T1–T2 breast cancer abstracted from the San Jose–Monterey registry. [file bcr934-S6.pdf]

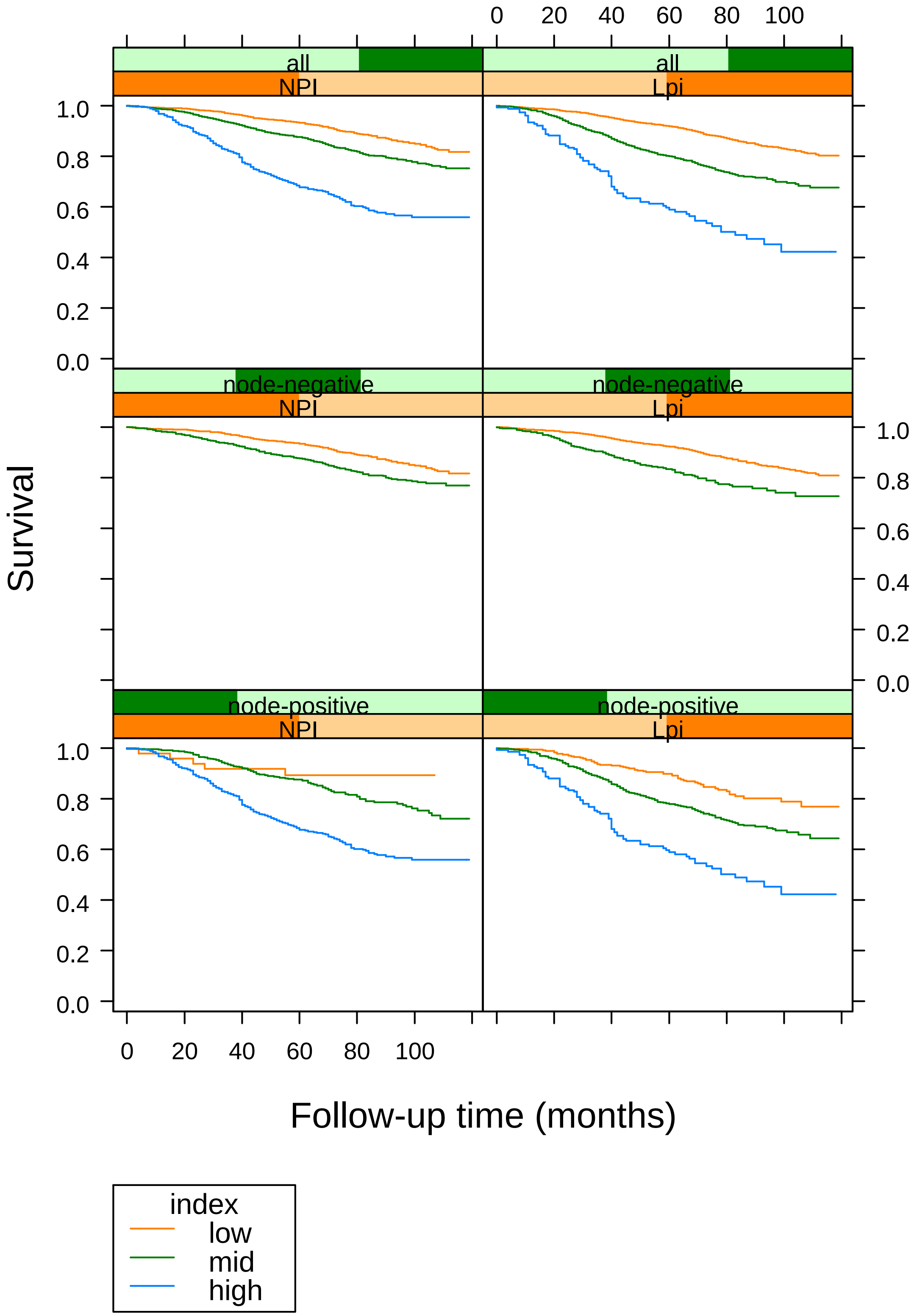
all
NPI
all
Lpi
node-negative
NPI
node-negative
Lpi
node-positive
NPI
node-positive
Lpi
Survival
Follow-up time (months)
0 20 40 60 80 100
1.0 0.8 0.6 0.4 0.2 0.0
index
low
mid
high

Additional file 6

Kaplan-Meier survival estimates for T1-T2 breast cancer abstracted from the San Jose-Monterey registry, as a function of the Nottingham Prognostic Index (NPI, left column) or the ratio-based index (*Lpi*, right column). From top to bottom: all cases (top, n=4204 patients), node-negative (middle, n=2961), and node-positive (bottom, n=1243).
