# Supplementary material for: Ratios of involved nodes in early breast cancer
Source: Breast Cancer Res. 2004 Oct 6;6(6):R680–8. doi: 10.1186/bcr934 (PMC1064081; doi:10.1186/bcr934)
Supplement: Additional File 7 — Text on ratios and the TNM categorization. [file bcr934-S7.doc]

# Additional file 7: Ratios and the TNM categorization

*Synopsis: Ratios are compared with the TNM nodal categorization, using data blinded to the original study. The comparison finds that the TNM performs well but can cause artefactual results inherent to categorization. Non-categorized ratios showed more stable results in diverse data samples.*

The manuscript investigated nodal numbers and ratios as continuous variables. Many questions might arise. What is the robustness of ratios, e.g. are they reliable or not in small datasets, as usually found in single institution studies. Should ratios be categorized or not. How they compare with more conventional categorizations of nodal involvement, e.g. the TNM [1,2]. How they compare with prognostic indexes, e.g. the Nottingham Prognostic Index (NPI) [3]. These are all complex issues that are actively investigated [4,5]. But, even though the questions cannot be addressed in a single paper, some form of answer is needed to evaluate whether or not ratios warrant further attention.

The data used in this Additional file was abstracted from the San Jose-Monterey (SJM) registry [6]. Data beginning from 1992 are available for the SJM and two other registries (Alaska and Los Angeles). These datasets were not incorporated in the manuscript's modeling. The SJM was chosen for its substantial but manageable size. Using the same manuscript's selection criteria, 4204 records were retrieved, representing 2961 node-negative and 1243 node-positive patients.

For the categorization of ratios, cutoffs of 20% and 50% were chosen for the proportion np/nx: np/nx=0, 0<np/nx≤20%, 20%<np/nx ≤50%, and np/nx>50%. The priority was given to simplicity and to a reasonable partition of the data (avoidance of too small categories, Table 1). The choice was set in view of a Danish study which argued for a 50% cutoff [7], and by the mnemonically attractive coincidence of the digits with tumor size cutoffs of 20 mm and 50 mm in the TNM classification [1].

Cutoffs of −3.5, −1 and 0 were chosen for the log-odds *L* (for recall, estimated as Log_e_[(np+0.5)/(nn+0.5)]): *L*≤−3.5, −3.5<*L*≤−1, −1<*L*≤0, *L*>0. The considerations were: *L*=−3.5 was the lowest boundary in node-positive cases (selection specified nx≤50), and was also the median in node-negative cases (Table 2); *L*=−1 was the highest boundary in node-negative cases, i.e. Log_e_(0.5/1.5); and *L*=0 corresponded to np=nn (i.e. np/nx=50%).

No attempt was made to "optimize" cutoffs for "best results". All considerations for cutoffs were irrelevant to the SJM itself and likewise the manuscript were blinded to the SJM data.

Ratios were compared with the TNM pathological nodal classification into 4 main categories: N0, np=0; N1, 0<np≤3; N2, 3<np≤9; N3, np>9. The statistical analyses used proportional hazard models, identical to the manuscript, differing only by the nodal variables. The end-point in the models was mortality from any-cause rather than breast-cancer mortality in view of the shorter follow-up in the SJM dataset. The Wald test was used to test the significance of single variables. The likelihood ratio test was used to test the joint contribution of the nodal variables [8]. Different subgroups were used to evaluate the stability of the comparisons:

- the full SJM dataset (results reported in Additional file 3);

- the node-positive cases (Additional file 4);

- in 1000 subsamples, each of 300 cases to simulate small datasets, randomly extracted with replacement from the SJM, irrespective of nodal status (Additional file 5);

- in 1000 subsamples, each of 300 cases, randomly extracted with replacement from the SJM node-positive cases (Additional file 6).

Additional files 3 and 4 show that the TNM nodal classification, and ratios, categorized or continuous, provided comparable R^2^_N_ values. However, the categorizations required two or three hazard ratios to arrive at the same result obtained with the proportion's or the log-odds' single hazard ratio. Moreover, the categorizations caused wider confidence intervals despite the large sample sizes, indicating that results based on the TNM or on categorized ratios might become unreliable in smaller datasets.

Additional file 5 shows the results that might have been expected if the prognostic role of nodal variables was investigated in small studies. The categorized variables appeared to provide a better model fit (larger R^2^_N_). However, this was at the expense of more "degrees of freedom", causing a loss of statistical power. The contribution of the categorized nodal variables to model fit was significant (at p-value 0.05) in only 599 to 626 of the 1000 subsamples. In other words, 40% of the multivariate studies performed with 300 patients would have been unable to demonstrate the statistical significance of nodal involvement. Additional file 5 also shows that the hazard ratio of N1 relative to N0 tested significant in only 250 simulation runs, while N2 relative to N0 tested significant in 492 simulation runs. That is, N1 vs. N0 (1-3 involved nodes vs. none involved) and N2 vs. N0 (4-9 involved nodes vs. none) would have been interpreted as showing "no significant effect" in 75% and in 51% of the studies, respectively. This is to be contrasted with the simple non-categorized proportion or log-odds which gave more stable results, with 70-75% of the runs testing significant.

Additional file 6 shows the same pattern as Additional file 5. The statistical power was improved (same subsamples size, but more events) resulting in more runs reaching significance. Nevertheless, the categorized nodal variables still compared less favorably, that is, globally fewer runs tested significant (74.4%-74.9% vs. 81.6%-83.3%), and, separately N2 vs. N1 reached significance in less than half of the runs (31.7%-43.9%).

Simulation runs were also done with absolute numbers of nodes. The performance was intermediary. Results were not recorded since these comparisons were redundant with the manuscript's already extensive evaluation of numbers vs. ratios.

In summary, the TNM nodal classification performed well in large samples. But results were unstable in small samples. Simulation runs clarified how categorization could cause artefactual "no effect" subgroups. This artefact occurred much less frequently with simple non-categorized ratios. Non-categorized ratios performed consistently in large and in small samples. Their results remained stable whether node-positive cases were analyzed separately or pooled with node-negative cases.

### References

1. Sobin LH, Wittekind CH (editors): *TNM Classification of Malignant Tumours*, 6th edn. New York: Wiley; 2002:131-141.

2. Greene FL, Page DL, Fleming ID, Fritz AG, Balch CM, Haller DG, Morrow M: *AJCC Cancer Staging Handbook. TNM Classification of Malignant Tumors*, 6th edn. New York: Springer Verlag; 2002:255-281.

3. Kollias J, Vernon-Roberts E, Blamey RW, Elston CW: **A simple index to predict prognosis independent of axillary node information in breast cancer: comment.** *Aust N Z J Surg* 1998, **68:**865-866.

4. Schemper M: **Predictive accuracy and explained variation.** *Stat Med* 2003, **22:**2299-2308.

5. Royston P, Sauerbrei W: **A new measure of prognostic separation in survival data.** *Stat Med* 2004, **23:**723-748.

6. National Cancer Institute. *Surveillance, Epidemiology, and End Results (SEER) Program (*www.seer.cancer.gov*) Public-Use Data (1973-2001), National Cancer Institute, DCCPS, Surveillance Research Program, Cancer Statistics Branch, released April 2004, based on the November 2003 submission.* Bethesda, MD: National Cancer Institute; 2004.

7. Rostgaard K, Mouridsen HT, Vaeth M, Holst H, Olesen KP, Lynge E: **A modified Nottingham prognostic index for breast cancer patients diagnosed in Denmark 1978–1994.** *Acta Oncol* 2001, **40:**838-843.

8. Therneau TM, Grambsch PM: *Modeling Survival Data: Extending the Cox Model*. New York, NY: Springer-Verlag; 2000:87-152.
